# Anti-Metastatic and Anti-Angiogenic Effects of Curcumin Analog DK1 on Human Osteosarcoma Cells In Vitro

**DOI:** 10.3390/ph14060532

**Published:** 2021-06-03

**Authors:** Muhammad Nazirul Mubin Aziz, Nurul Fattin Che Rahim, Yazmin Hussin, Swee Keong Yeap, Mas Jaffri Masarudin, Nurul Elyani Mohamad, Muhammad Nadeem Akhtar, Mohd Azuraidi Osman, Yoke Kqueen Cheah, Noorjahan Banu Alitheen

**Affiliations:** 1 Department of Cell and Molecular Biology, Faculty of Biotechnology and Biomolecular Sciences, Universiti Putra Malaysia, Serdang 43400, Selangor, Malaysia; muhammadnazirulmubin@gmail.com (M.N.M.A.); nurulfattincherahim@gmail.com (N.F.C.R.); yazminh93@gmail.com (Y.H.); masjaffri@upm.edu.my (M.J.M.); elyani.mohamad@gmail.com (N.E.M.); azuraidi@upm.edu.my (M.A.O.); 2 China-ASEAN College of Marine Sciences, Xiamen University Malaysia, Sepang 43900, Selangor, Malaysia; skyeap@xmu.edu.my; 3 UPM-MAKNA Cancer Research Laboratory, Institute of Bioscience, Universiti Putra Malaysia, Serdang 43400, Selangor, Malaysia; 4 Biotechnology Research Institute, Universiti Malaysia Sabah, Kota Kinabalu 88400, Sabah, Malaysia; 5 Department of Chemistry, Ghazi University, Dera Ghazi Khan 32200, Pakistan; nadeemupm@gmail.com; 6 Department of Biomedical Science, Faculty of Medicine and Health Science, Universiti Putra Malaysia, Serdang 43400, Selangor, Malaysia; ykcheah@upm.edu.my

**Keywords:** curcumin analog DK1, human osteosarcoma (U-2 OS, MG-63), metastasis, angiogenesis

## Abstract

Osteosarcoma (OS) is a life-threatening malignant bone tumor associated with poor prognosis among children. The survival rate of the patient is still arguably low even with intensive treatment provided, plus with the inherent side effects from the chemotherapy, which gives more unfavorable outcomes. Hence, the search for potent anti-osteosarcoma agent with promising safety profile is still on going. Natural occurring substance like curcumin has gained a lot of attention due to its splendid safety profile as well as it pharmacological advantages such as anti-metastasis and anti-angiogenesis. However, natural curcumin was widely known for its poor cellular uptake, which undermines all potential that it possesses. This prompted the development of synthetically synthesized curcuminoid analog, known as (Z)-3-hydroxy-1-(2-hydroxyphenyl)-3-phenylprop-2- en-1-one (DK1). In this present study, in vitro scratch assay, transwell migration/invasion assay, HUVEC tube formation assay, and ex vivo rat aortic ring assays were performed in order to investigate the anti-metastatic and anti-angiogenic potential of DK1. For further comprehension of DK1 mechanism on human osteosarcoma cell lines, microarray gene expression analysis, quantitative polymerase chain reaction (qPCR), and proteome profiler were adopted, providing valuable forecast from the expression of important genes and proteins related to metastasis and angiogenesis. Based on the data gathered from the bioassays, DK1 was able to inhibit the metastasis and angiogenesis of human osteosarcoma cell lines by significantly reducing the cell motility, number of migrated and invaded cells as well as the tube formation and micro-vessels sprouting. Additionally, DK1 also has significantly regulated several cancer pathways involved in OS proliferation, metastasis, and angiogenesis such as PI3K/Akt and NF-κB in both U-2 OS and MG-63. Regulation of PI3K/Akt caused up-regulation of genes related to metastasis inhibition, namely, PTEN, FOXO, PLK3, and GADD45A. Meanwhile, NF-κB pathway was regulated by mitigating the expression of NF-κB activator such as IKBKB and IKBKE in MG-63, whilst up-regulating the expression of NF-κB inhibitors such as NFKBIA and NFKBIE in U-2 OS. Finally, DK1 also has successfully hindered the metastatic and angiogenic capability of OS cell lines by down-regulating the expression of pro-metastatic genes and proteins like MMP3, COL11A1, FGF1, Endoglin, uPA, and IGFBP2 in U-2 OS. Whilst for MG-63, the significantly down-regulated oncogenes were Serpin E1, AKT2, VEGF, uPA, PD-ECGF, and Endoglin. These results suggest that curcumin analog DK1 may serve as a potential new anti-osteosarcoma agent due to its anti-metastatic and anti-angiogenic attributes.

## 1. Introduction

Osteosarcoma (OS) has been reported as one of the most common cancers among children and adolescent, with a global incidence rate ranged between 3 and 4.5 cases per million [1,2]. This alarming event also hit Malaysia, with approximately 3% from a total of 800 children were diagnosed with this particular cancer type [3]. Additionally, OS was also found to occur mainly among boys with indefinite symptoms such as swelling as well as strain related pain for average of 3 to 4 months before manifesting more visible signs of OS like erythema, brittle bones, and tumor formation [4,5,6]. Despite the advanced and intensive treatments applied such as surgery together with chemotherapy, the five year survival’s rate of the patient dropped significantly to 30% once diagnosed with metastatic OS [7]. Evidently, this aggressive metastatic OS in concert with angiogenesis further foster the tumor growth and metastasis occurrence, which often results in pulmonary metastasis [8]. On top of that, chemotherapy drug that is commonly used like Doxorubicin also poses appalling side effects including cardiotoxicity, myelosuppression, and mucositis when administered at a high dosage [9,10,11]. Thus, younger patient such as children are more susceptible to these side effects, which then tremendously reduce their survival rate during or after the treatment [12]. Hence, identification of new anti-osteosarcoma agents derived from natural products that display excellent anti-metastatic and anti-angiogenic potential like curcumin is imperative [13].

Curcumin is a natural bioactive compound derived from a spice called turmeric, originated from the rhizomes of *Curcuma Longa* plant [14]. This spice can be widely found in most Asian countries and has been used for centuries either as food condiments or served as medicinal remedies [13]. This active compound was prominently known for its wide spectrum of pharmacological activities including anti-oxidant, anti-inflammatory, anti-proliferation, and anti-metastatic properties [13,14]. In addition to these exceptional qualities, curcumin also has a good safety profile even at high dosage. However, due to its low solubility, and thus poor oral absorption that affects curcumin’s efficacy, further improvement is required to overcome this limitation [14,15]. For instance, poor uptake of dietary curcumin by the cells was contributed by too many hydrogen bonding motifs existing in the curcumin structure, which in turn causes an intransigent interaction with phospholipids bilayer of the cells [16]. This event will immensely restrict the diffusion of dietary curcumin to the cytoplasm which one of the main target areas for curcumin to manifest its bioactivities [16]. Therefore, for the past few years many synthetically synthesized curcumin analogs with improved physiological properties have shown better cytotoxic and anti-metastasis effects as well as anti-angiogenesis activities which have elevated curcumin’s potential as an anti-cancer agent [17,18].

This present study employed a curcumin analog namely (Z)-3-hydroxy-1-(2-hydroxyphenyl)-3-phenylprop-2-en-1-one (DK1) with simplified structure and optimal hydrogen bonding in order to tackle the shortfall in curcumin [19,20]. Moreover, DK1 has also been proven to exhibit better cytotoxic activity, cell cycle dysregulation and apoptosis induction in several cancer cell lines such as MCF-7 breast cancer cell line, HT29, and SW620 colon cancer cell lines as well as U-2 OS and MG-63 osteosarcoma cell lines [19,21,22]. Even though DK1 showed promising anti-cancer effect towards several cancer cell lines, no study has been reported on its anti-metastatic and anti-angiogenic capabilities. Therefore, the primary aim of this study was to investigate the in vitro anti-metastatic and anti-angiogenic capability of curcumin analog DK1 together with the underlying mechanisms, which results in an utmost evaluation of DK1 effectiveness as an anti-osteosarcoma agent so far.

## 2. Results

### 2.1. Curcumin Analog DK1 Inhibit Motility of Both OS Cell Lines

**Figure 1:**
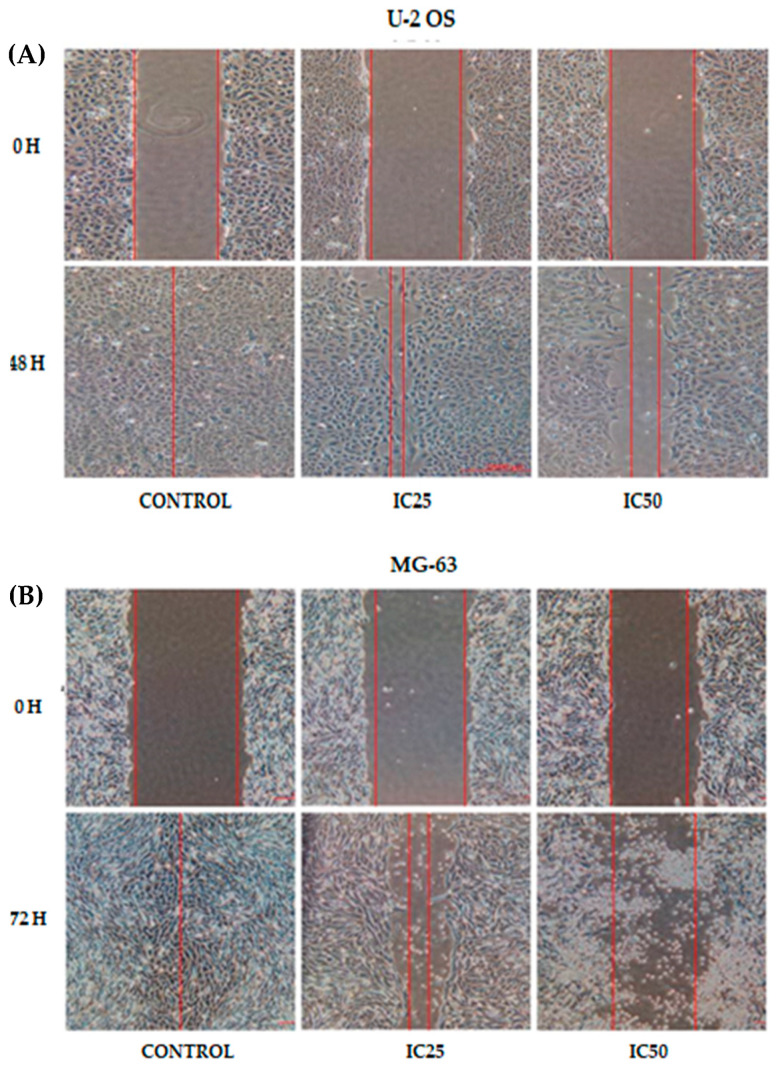

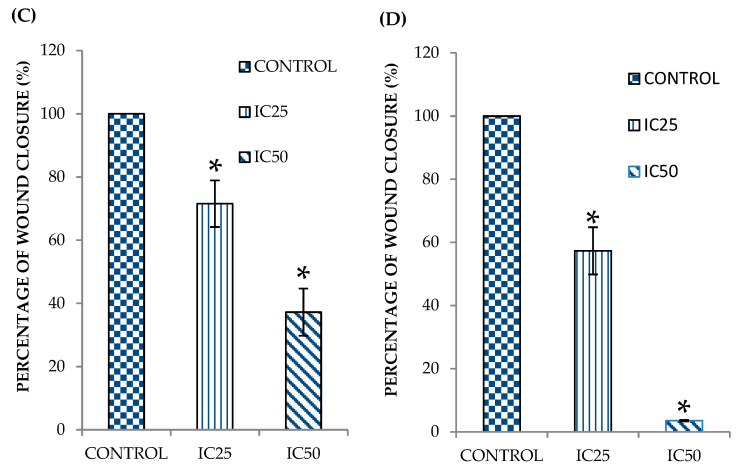
(**A**,**B**) Images showing wound closing activity of U-2 OS and MG-63 cell lines treated with two different concentrations (IC_25_, IC_50_) of DK1. U-2 OS untreated group required 48 h to completely heal the wound, while for MG-63, 72 h were needed to close the gap. (**C**,**D**) Percentage of wound closure for both U-2 OS and MG-63 cell lines when a scratch was introduced. Each experiment was done in triplicate and all data were expressed as mean ± standard error mean (S.E.M). * *p* < 0.05 compared with corresponding control. (Magnification: 100×).

To further evaluate curcumin analog DK1 potential as an anti-metastatic agent, scratch assay was conducted for both U-2 OS and MG-63 osteosarcoma cell lines treated with DK1. This assay provides an insight of the DK1 efficacy by examining the advancement of the wound edge or cells recolonization at the wound areas [23]. Two different inhibition concentrations (IC_25_ and IC_50_) of the DK1 were used to treat both cell lines onset before evaluating the motility rate. Figure 1A,C shows that DK1 has significantly decreased the U-2 OS percentage of wound closure to 37.2 ± 7.4% in IC_50_ treated group compared with 100% from the control group. Similar pattern was observed in Figure 1B,D for MG-63 whereby there was a significant reduction in IC_50_ treated group (3.5 ± 0.2%) as compared to the control group (100%). The statistical results suggest that curcumin analog DK1 interfered with migratory activity of both osteosarcoma cell lines in a dose dependent manner.

### 2.2. Curcumin Analog DK1 Inhibit Migration and Invasion Capability of Both OS Cell Lines

**Figure 2:**
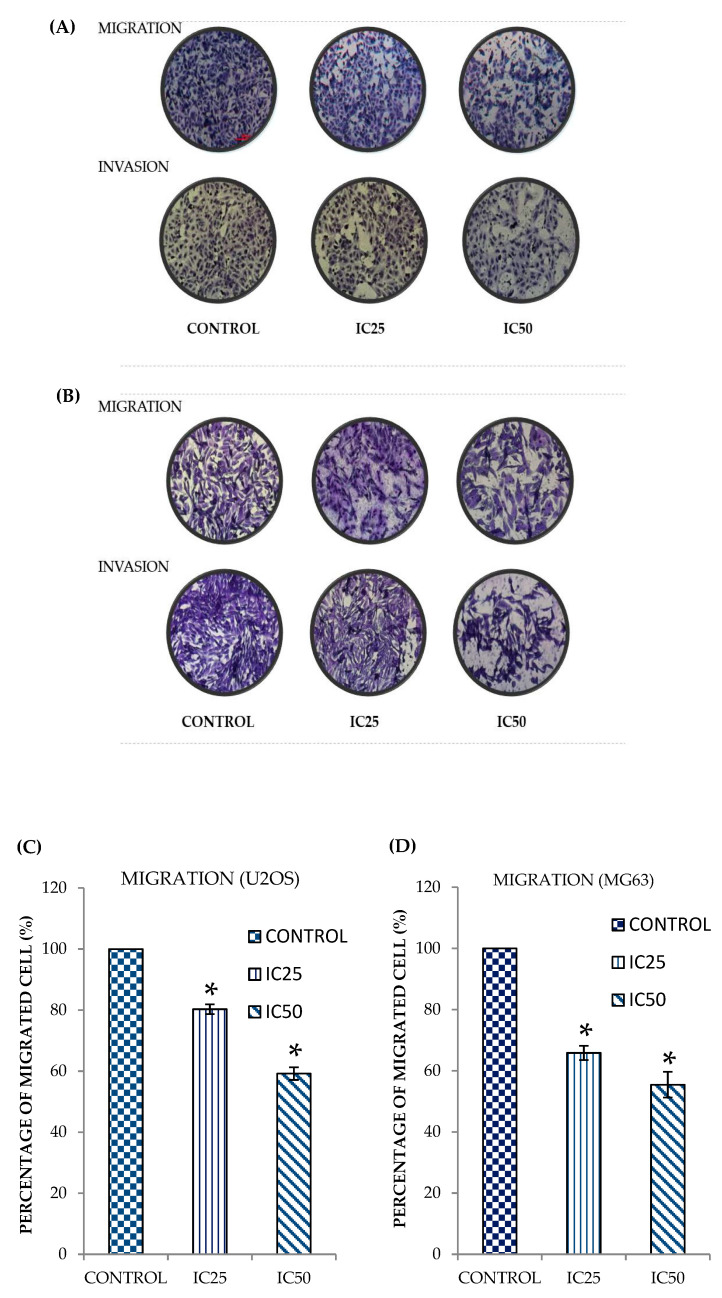

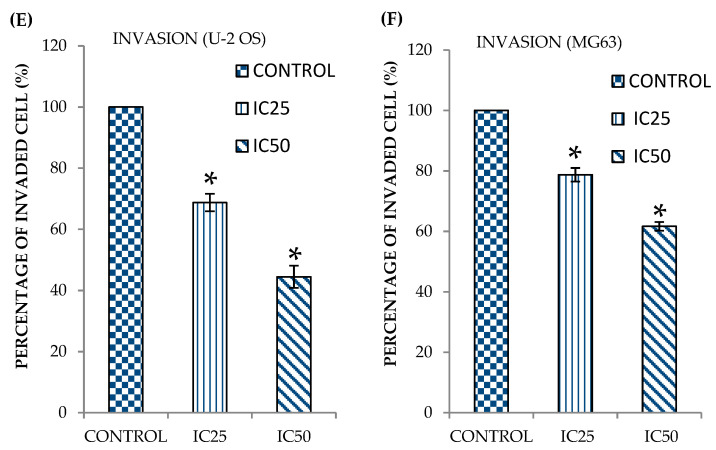
Representative images of transwell migration/ invasion assays for both (**A**) U-2 OS and (**B**) MG-63 after being treated with two different concentrations (IC_25_, IC_50_) of DK1 for 48 h. For migration assay the cells were required to migrate through membrane with 8 µM pore-size. While for invasion assay the cells were allowed to invade the same pore-size membrane coated with matrigel. (**C**,**D**) Quantification analysis of U-2 OS and MG-63 based on the percentages of migrated cell treated with DK1. (**E**,**F**) Percentages of invaded cell for both U-2 OS and MG-63 treated with DK1. All experiments were done in triplicate and all data were expressed as mean ± standard error mean (S.E.M). * *p* < 0.05 compared with corresponding control. (Magnification: 100×).

Metastasis is a very complex and systematic process that depends on several pivotal steps such as cells migration and invasion [24]. Hence, the search for anti-metastatic agent that capable to impede these processes is absolutely vital. In order to determine whether curcumin analog DK1 possess these crucial attributes, both transwell migration and invasion assays were performed in this study. As shown in Figure 2A (U-2 OS) curcumin analog DK1 has substantially decreased the number of migrated and invaded cells in a dose-dependent manner These observations were also reflected in the quantification analysis depicted in Figure 2C,E, where the percentage of migrated and invaded cells were significantly reduced to 59.1 ± 2.0% and 44.4 ± 3.6%, respectively. Expectantly, similar result also was observed in MG-63 treated with DK1 (Figure 2B), where the number of migrated and invaded cells were notably declined with the increment of DK1 concentration. Figure 2D,F substantiates that in IC_50_ treatment group the percentage of both migrated and invaded cells in MG-63 cell lines were significantly decreased to 55.4 ± 4.2% and 61.6 ± 1.4%, respectively.

### 2.3. Curcumin Analog DK1 Possesses Anti-Angiogenesis effects

**Figure 3:**
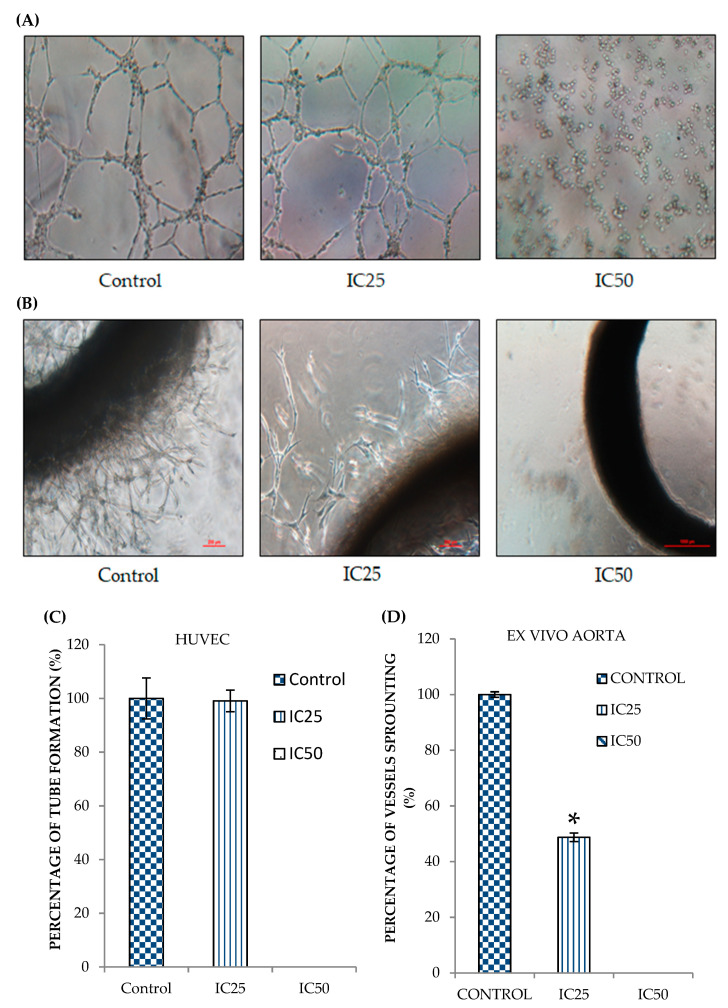
Curcumin analog DK1 inhibits the tubes formation as well as the micro-vessels sprouting; ultimately served as potential evidence as anti-angiogenic agent. (**A**) Images represent HUVEC tube formation assay when treated with two different concentrations (IC_25_, IC_50_) of DK1 (Magnification: 100 ×). (**B**) Images of ex vivo rat aorta ring assay when exposed to two different concentrations (IC_25_, IC_50_) of DK1 (Magnification: 100×). (**C**) Percentage of tubes formation when treated with two different concentration of DK1. (**D**) Percentage of micro-vessels sprouting of ex vivo rat aorta when treated with two different concentrations of DK1. Both experiments were done in triplicate and all data were expressed as mean ± standard error mean (S.E.M). * *p* < 0.05 comparing with corresponding control.

Angiogenesis is one of the key hallmarks in cancer metastasis; essentially aid in fostering the tumor growth at a distant site [25]. To delineate the anti-angiogenic potential possessed by curcumin analog DK1, two important angiogenic assays in particular HUVEC tube formation and Ex Vivo Ring AORTA assays were conducted. Human umbilical vein endothelial cells (HUVECs) were seeded on top of matrigel and once tubulogenesis activity was observed, the tubes formed were analyzed by comparing the DK1 treated groups with untreated control group [26]. As depicted in Figure 3A,C the number of tubes formation were diminished significantly as the concentration of the DK1 increased up to IC_50_ concentration. To further evaluate as well as to confirm the anti-angiogenesis activity of the DK1, ex vivo rat aorta ring assay was performed. Aorta ring assay was widely used in many studies since it is capable to mimic micro-vessels formation related to angiogenesis activity in vivo by encompassing the early stage of vessel sprouting, matrix remodeling as well as vessels lumenization [27]. Based on Figure 3B,D similar pattern also can be observed where curcumin analog DK1 notably reduced the micro-vessels outgrowth from the fragmented thoracic aorta of the rat. These results suggested that curcumin analog DK1 posed anti-angiogenic potential in a dose dependent manner.

### 2.4. Curcumin Analog DK1 Induce Several Cancer-Related Pathways in Both OS Cell Lines

**Figure 4:**
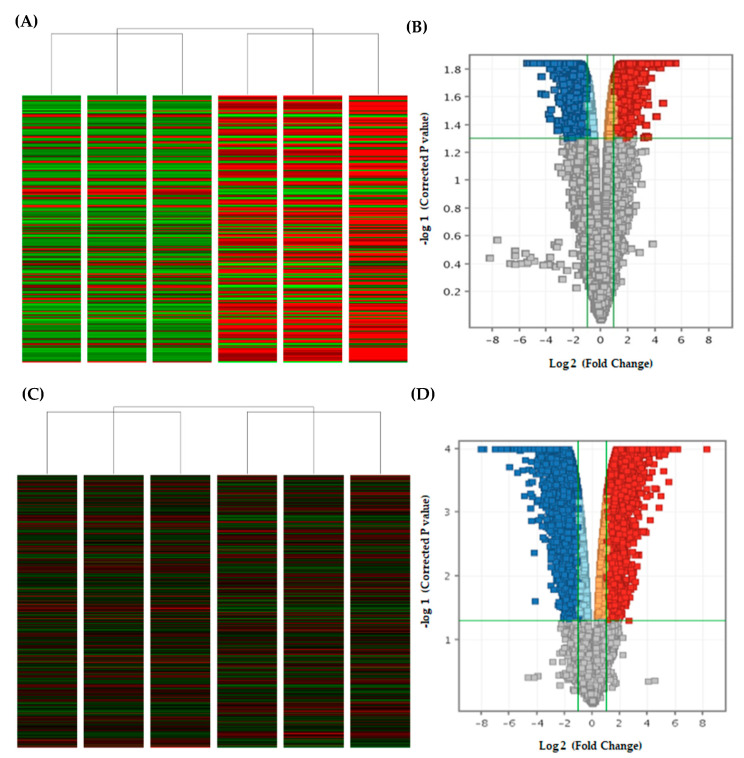
Images above indicate the differential expression of regulated genes in both U-2 OS and MG-63 osteosarcoma cell lines between control group and DK1 IC_50_ treated group. (**A**,**C**) Heat maps of differential expression for both U-2 OS and MG-63 cell lines; interpret the regulated genes expression in pattern visualization form when clustering similar treatment groups together. Each grid in row represents regulated genes, while each column served as treatment group samples. (**B**,**D**) Volcano plots of differential expression of regulated mRNA for both U-2 OS and MG-63 with *p* < 0.05 and fold change ≥ 2 as well as ≤ −2. Blue color (down-regulated) indicates that the regulated genes have *p* < 0.05 with ≤ −2 fold changes. While Red color (up-regulated) indicates that the expression of regulated genes have *p* < 0.05 with fold changes ≥ 2.

**Figure 5:**
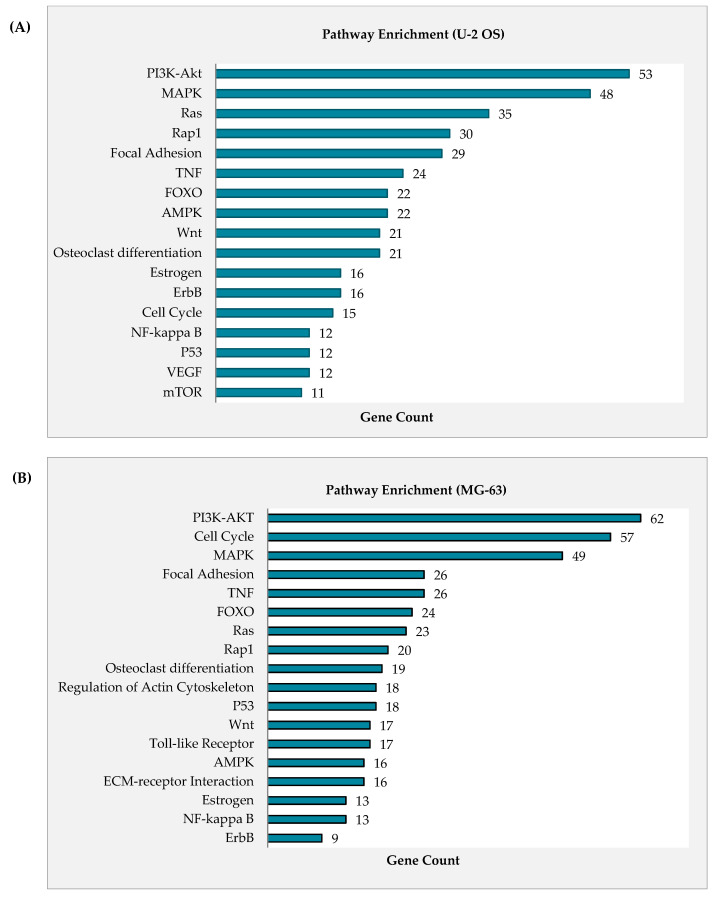
(**A**,**B**) Exhibited the enrichment pathway in cancer that is associated to the regulated expression genes for both U-2 OS and MG-63 osteosarcoma cell lines. All characterized pathways were selected based on the gene count numbers with *p* value < 0.05.

Osteosarcoma is a relatively heterogeneous cancer type that hampers the cancer medicine with different prognosis and even response towards chemotherapy [28,29]. Therefore, rather than focusing on with specific oncogenes, understanding and characterizing the molecular mechanism such as cancer pathway as a whole will effectively rectify against this genetic diversity as well as providing accurate insight on the effectiveness of novel anti-cancer agent like curcumin analog DK1 [30]. Microarray was used to assess the differential expression of regulated mRNA between untreated control group with DK1 IC_50_ treated group. Figure 4A–D represent the heat maps and volcano plots from the microarray transcriptome analysis. These results mainly used for visualization of the expression pattern and identification of significantly regulated mRNA in both U-2 OS and MG-63 with *p* value less than 0.05 and fold changes greater than or equal to 2 as well as less than or equal to −2 [31]. From the analysis, there were 2081 up-regulated and 2150 down-regulated mRNA in U-2 OS, while 3793 up-regulated and 3768 down-regulated mRNA in MG-63. Figure 5A,B illustrates the pathways in cancer that are regulated in both U-2 OS and MG-63 when treated with curcumin analog DK1. Highly enriched pathway was determined based on the gene count numbers mapped into it and for instance PI3K-Akt was the most enriched pathway in both U-2 OS and MG-63 osteosarcoma cell lines with 53 and 62 gene counts, respectively. To further confirm the anti-metastatic capability of curcumin analog DK1, Table 1 and Table 2 show the summarized list of regulated genes that involve either in promoting cell metastasis or results in inhibitory activity in both U-2 OS and MG-63 cell lines, respectively.

### 2.5. Curcumin Analog DK1 Regulates Several Genes and Protein Related to Metastasis and Angiogenesis

**Figure 6:**
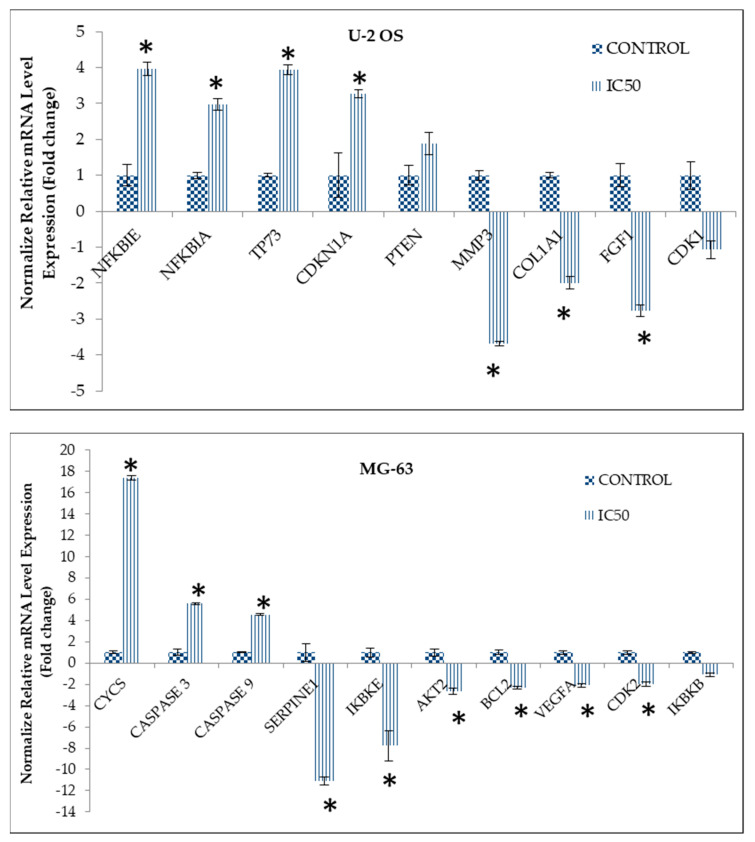
The differential expression of regulated genes in both U-2 OS (**top**) and MG-63 (**down**) were validated using quantitative real-time PCR (qPCR). All validated genes were mostly involved either in metastasis or angiogenesis process in osteosarcoma cell lines treated with DK1 (IC_50_) for 48 h. The expressions of all genes were normalized to GUSB and ACTB housekeeping genes. The analysis were done in triplicate and expressed as mean ± standard error mean (S.E.M). The significant of the data was set at * *p* < 0.05 and fold changes more than 2 which compared between untreated control group and the DK1 treated group.

In this study, quantitative real-time PCR (qPCR) assay and proteome profiler were conducted primarily to validate the effect of curcumin analog DK1 towards expression of metastasis related genes and proteins for both osteosarcoma cell lines. Based on Figure 6, DK1 substantially increased the expression of genes that were involved in metastatic inhibition in U-2 OS cell lines such as NFKBIE, NFKBIA, and TP73. Furthermore, similar occurrence also can be observed in MG-63 where several anti-metastatic genes, namely, Cycs, Caspase 3, and Caspase 9 were significantly increased when treated with DK1 (IC_50_). On top of that, DK1 was also capable to regulate pro-metastatic genes in both U-2 OS and MG-63 osteosarcoma cell lines; for instance, MMP3, COL1A1, and FGF expression were significantly down-regulated in U-2 OS. Whilst in MG-63, pro-metastatic genes that notably down-regulated were Serpin E1, AKT2, VEGF, IKBKE, and BCL-2. Table 3 further corroborates the DK1 anti-metastatic potentials toward osteosarcoma cell lines in a protein expression level which pro-metastatic proteins like Endoglin, uPA, IGFBP, and FGF were significantly down-regulated in U-2 OS, while in MG-63 such event also can be observed where the expression of pro-metastatic proteins such as Serpin E1, Endoglin, IGFBP2, and PD-ECGF were decreased significantly.

## 3. Discussion

Curcumin is a natural occurring substance that has been recognized as safe by US Food and Drug Administration (FDA). Many studies have utilized this compound especially in clinical trials for cancer therapies, which involved several types of cancer including colorectal cancer, breast cancer, and lung cancer [32,33]. Unfortunately, natural curcumin has limited therapeutics potential which mainly contributed by the poor bioavailability in vivo [33]. Due to this circumstance, countless attempts have been done with an ultimate aim to improve this physiological famine as well as the biological activities [33,34]. Improvement of natural curcumin via synthetic analog or derivative has emerged steadily over the years, one of the alternative examples was curcumin analog DK1. This lower molecular weight analog has simplified shorter chains structure and optimal hydrogen bonding motifs differs from dietary curcumin [19,20]. These changes have significantly improved the cellular uptake of the DK1 analog as proved by natural curcumin requiring higher dosages utilization in human colon carcinoma cell lines in order to exhibit 50% inhibition concentration compared to DK1 [22]. Moreover, similar pattern can be observed in human osteosarcoma cell lines treated with natural curcumin where it required 72 h incubation time in an attempt to pose 50% inhibition concentration, whilst DK1 only needed 48 h incubation time to accomplish similar efficacy [21]. Apart from having better cytotoxicity effects in several human cancer cell lines, curcumin analog DK1 also demonstrated that it is capable to induce apoptosis and dysregulate the cell cycle activities of several human cancer cell lines such as MCF-7, HT29, SW620, U-2 OS, and MG-63 [19,21,22]. With all these promising aspects shown by the DK1, further evaluations were essential especially on the anti-metastasis and anti-angiogenesis effects of this particular analog. 

Metastasis remains the most challenging aspects in OS management with only 30% survival rates among the diagnosed patients [7]. For DK1 to be conveyed as a potential anti-osteosarcoma agent it ought to demonstrate anti-metastatic and anti-angiogenic capabilities against both human osteosarcoma cell lines. For this particular study two different OS cell lines were used namely U-2 OS and MG-63. Both cell lines have distinct dissimilarity in term of aggressiveness where U-2 OS is capable to migrate and invade at a higher rate in contrast with MG-63 [35]. One of the indispensable steps in metastasis is cell migration; this process was driven by continuous actin polymerization-mediated membrane protrusion and interaction with extracellular matrix (ECM) which then allowed the cells to move from one site to another [36,37]. Initially, in order for metastasis process to be commenced, the cancer cells need to be able to invade surrounding tissues by adhering to the ECM and initiate proteolytic degradation then propel itself further into lymphatic or blood vessel [36]. This study revealed that curcumin analog DK1 significantly inhibited the cell motility for the wound closure in a concentration-dependent manner for both U-2 OS and MG-63 cell lines. Moreover, this pattern also can be perceived in the in vitro transwell migration and invasion assays, where DK1 substantially reduced the percentage of migrated and invaded cells in both human osteosarcoma cell lines. The promising use of DK1 as anti-osteosarcoma agent is further accentuated by the anti-angiogenic effects that it possesses. Angiogenesis is one of the key modulators in cancer metastasis; where it forms new blood vessels that co-opt from pre-existing one which enable the supplies of oxygen, growth factors, and nutrients that necessary for advancement of metastasis including act as a means for the tumor dissemination at secondary sites [25,38,39]. As demonstrated in this study, DK1 significant inhibition of tube formation ability as well as impediment of the micro-vessels sprouting was observed in a dose-dependent manner. These results proposed that curcumin analog DK1 is capable to serve as a potential anti-osteosarcoma agent since it capable to inhibit the migration and invasion abilities in both osteosarcoma cell lines as well as obstruct the angiogenesis process proven by significant reduction in both HUVEC tube formation and micro-vessels sprouting. Furthermore, these results also suggest that even with simplified structure, curcumin analog DK1 was still capable to manifest potent anti-metastasis and anti-angiogenesis potential in vitro which mainly imparted by retaining the ꞵ-diketo moiety similar to the other curcumin analog such as DMC and 2-hydroxycurcuminoids [40]. These analogs reportedly exhibited potent anti-cancer potential in SW620 colon cancer and MCF7 breast cancer respectively [40].

Determination of regulated cancer pathways by DK1 is more efficient when using microarray analysis. The most significant and highly enriched pathway in U-2 OS and MG-63 osteosarcoma cell lines treated with DK1 was PI3K-Akt with 53 and 62 gene count numbers, respectively. PI3K/Akt is one of the highly activated cancer pathways in osteosarcoma, promoting the OS proliferation, metastasis, angiogenesis, and even tumorigenesis [41]. Activation of this tightly regulated pathway involves multistep process that starts with receptor activation to trigger PI3k expression which subsequently promotes Akt phosphorylation [41]. Akt plays a central role in downstream cascade like the activation of proto-oncogenes, such as NF-κB, Wnt, MMP, BCL-2, and VEGF, as well as suppressing tumor-suppressor genes like FOXO1, Bax, Caspase, and Cycs [41]. Data obtained suggested that curcumin analog DK1 has the ability to regulate PI3K/Akt pathway by up-regulating the expression of PTEN in both OS cell lines; this gene is a prominent negative regulator of PI3k/Akt signaling pathway by targeting the PIP3 that notably known to act as Akt activator [41]. With significant up-regulated expression of PTEN induced by DK1, one of the Akt isoform especially Akt 2 was significantly down-regulated in both U-2 OS and MG-63. Moreover, DK1 also enhanced the expression of several other tumor suppressor genes that are related to this pathway such as FOXO, TP73, CDKN1A, Caspase, CYCS, and GADD45A while impeded the expression of other proto-oncogenes namely PIK3R3, BCL-2, IGFBP2, HGF, and FGF [41,42,43,44]. Another interesting pathway that has been regulated by DK1 was NF-κB pathway. NF-κB is one of the main molecules to be targeted by natural curcumin [45]. Cumulatively, nearly 7000 studies have reported about this inverse connection between curcumin and NF-κB with one of the earliest reports in 1995 [46,47]. Activation of NF-κB has been associated with enhancement of OS proliferation, metastasis, and angiogenesis including the suppression of apoptotic activity [48,49]. Multifactorial activation of NF-κB causes inhibitor like IκBs remain inactive and being degraded to release this molecule for translocation to the nucleus and aid in transcription of downstream target genes which consequently causes the advancement of metastatic OS [41,49]. According to the results, curcumin analog DK1 was capable to regulate this pathway by significantly up-regulating the expression of two important negative regulators for NF-κB in U-2 OS which were NFKBIA and NFKBIE, while in MG-63 treated with DK1 showed to significantly down-regulate the expression of two activator molecules of NF-κB which were IKBKB and IKBKE [48]. Evidently, microarray results also revealed that DK1 was capable to regulate one of the prevalent tumorigenesis signaling pathways, known as MAPK/ERK [50]. This pathway plays a pivotal role in cancer progression especially in the metastasis and angiogenesis processes in several cancer types including osteosarcoma [51]. DK1 regulates this signaling cascade by significantly up-regulating the expression of two prominent MAPK/ERK negative regulators, namely, DUSP 2 and DUSP 10 in both U-2 OS and MG-63 human osteosarcoma cell lines [52,53]. DUSP 2 is a negative regulator that preferentially regulates MAPK pathway by dephosphorylating ERK and p38 [52]. Whereas DUSP 10 was capable to regulate all three main groups of MAPK signaling cascade including JNK, p38 and ERK [53].

Considering OS heterogeneity, a slight variation in the expression of regulated genes or proteins between OS cell lines were expected [29]. For instance, these differences can be observed in Table 3; where MG-63 treated with DK1 significantly down-regulated the expressions of several oncogenes namely VEGF, PD-ECGF, and Endostatin differ from U-2 OS. Next, similar regulated proteins associated to metastasis and angiogenesis in both cell lines were further evaluated with the aim to strengthen the DK1 potential. As depicted in the results, curcumin analog DK1 efficiently down-regulated the protein expression of fibroblast growth factor (FGF) in both OS cell lines; FGF is known to promote various biological process in OS development including cells proliferation, invasion, migration, and angiogenesis [54]. Moreover, as evidenced by proteome profiler DK1 successfully down-regulated the protein expressions of both uPA and its inhibitor Serpin E1 in both OS cell lines; these two oncogenes have paradox interaction that is essential for promoting the cancer migration, invasion, and angiogenesis [55]. Endoglin protein expression also has been substantially down-regulated in both U-2 OS and MG-63 cell lines when treated with DK1; this protein plays a vital role in angiogenesis process when its response to hypoxic condition produced by the inhibition of another angiogenic related pathway such as VEGF [56]. Last, but not least, IGFBP-2 protein expression also has been significantly down-regulated in both OS cell line when exposed to DK1; this molecule serves as a carrier that binds to IGF and extends the IGF’s half-life in order to maintain the modulation, circulation, and concentration which essential for cancer development [57]. IGFBP-2 has been reported to involve in cancer metastasis, angiogenesis, and tumorigenesis [57].

## 4. Materials and Methods 

### 4.1. Preparation of Curcumin Analogue DK1

Curcumin analogue (Z)-3-hydroxy-1-(2-hydroxyphenyl)-3-phenylprop-2-en-1-one (DK1) was procured from Dr. Nadeem Akhtar from Universiti Malaysia Pahang who synthesized the DK1 following the outlined protocol published by Ali et al., 2017 [19].

### 4.2. Cell Treatment

Curcumin analogue DK1 was dissolved in 0.1% DMSO to make it solubilize in water [21]. Based on MTT assay results published by Aziz et al., 2018, three doses of curcumin analogue DK1 at 48 hours’ time-point were used throughout this study. The doses used to administer to U-2 OS were IC_75_ (30 µM), IC_50_ (19.6 µM), IC_25_ (2.2 µM) and for MG-63; IC_75_ (30 µM), IC_50_ (23.8 µM), IC_25_ (6.6 µM), respectively. 

### 4.3. In Vitro Scratch Assay

This assay was conducted by following the outlined protocol published by Liang et al., 2007 [58]. Both U-2 OS and MG-63 osteosarcoma cell lines were seeded in 6-well plate overnight to full confluency. By using a sterile yellow tip, a scratch was introduced in the middle of the well and the old media was discarded which then replaced with a fresh media containing different concentration of curcumin analogue DK1. The migration rate was observed up to 72 h using an inverted microscope (Nikon FC-35DX, Tokyo, Japan). The selected areas of the wound that were photographed during the initial phase of the experiment were labelled with a small dot with a fine needle permanent marker to ensure that the same areas were monitored throughout this study. Formula stated below was used to calculate the migration rate of the cells move towards the center of the wound:

(1) $$\mathrm{Percentage}\;\mathrm{of}\;\mathrm{wound}\;\mathrm{closure}\;=\;\frac{\mathrm{Area}\;\mathrm{of}\;\mathrm{wound}\;\mathrm{at}\;0h-\mathrm{Area}\;\mathrm{of}\;\mathrm{wound}\;\mathrm{at}\;(n)h}{\mathrm{Area}\;\mathrm{of}\;\mathrm{wound}\;\mathrm{at}\;0h}\times 100\%$$

### 4.4. In Vitro Transwell Migration and Invasion Assay

This transwell migration and invasion assays were conducted based on the presumption that the human osteosarcoma cell lines able to migrate or invade with the presence of stimulant. This assay was attempted by following the protocol published by Chen., 2005 [59]. For the invasion assay, 650 µl of matrigel was loaded to the upper chamber, meanwhile for the migration assay the upper chamber was not coated with any extracellular basement membrane. The basement membrane preparation was conducted by diluting the matrigel to a ratio 1:2 with a serum-free media and incubated at 37 °C for 2 h to ensure the membrane was completely solidified. The human osteosarcoma cell lines were serum-starved for 24 h before being seeded into the upper chamber coated with solidified matrigel at a density of 6 × 10^4^ cells/mL. For the lower chamber, 2 mL of fresh media supplemented with 10% FBS and several different concentrations of curcumin analogue DK1 was added. The inserts were kept in a 37 °C CO_2_ incubator for 48 h. Next, the non-migrated/invaded cells from the upper chamber were removed using a cotton swab; while migrated/invaded cells from the lower part of the upper chamber were fixed for 30 min in 1 mL of methanol before continuing with staining of the inserts using 0.5% of crystal violet. Later the inserts were photographed using an inverted microscope (Nikon FC-35DX, Tokyo, Japan) and analyzed.

### 4.5. In Vitro HUVEC Tube Formation Assay 

Fifty microliters of undiluted matrigel was used to coat the 96-well plate for 2 h. Next, the HUVEC cells were trypsinized and washed three times with PBS before being seeded to the pre-coated 96-well plate. Then HUVEC cells at a density of 1 × 10^5^ cells/mL were added together with different concentrations of DK1. After 10 h of incubation in 37 °C CO_2_ incubator, the tubes formed between the HUVEC cells were captured using an inverted microscope (Nikon FC-35DX, Tokyo, Japan). The analysis was performed by counting the number of branches with a minimum of three connected tubes. The percentage of tube formation was calculated by comparing the number of branches in DK1 treated group with untreated control group.

### 4.6. Ex Vivo Rat Aortic Ring Assay

The dorsal aorta was isolated from male Sprague-Dawley rats aged 5–7 weeks old. Under sterile condition the aorta was rinsed and washed three times with ice cold PBS before cut into ~1 to 1.5 mm pieces. Next the sections were placed on top of the matrigel-precoated 96-well plate before layered with another 50 µL matrigel and formed a sandwich-like structure. Once the top layer matrigel has completely solidified, fresh EGM media supplemented with different concentration of DK1 were added to the wells. The aorta was kept in 37 °C CO_2_ incubator for 10 days before being photographed. The analysis was conducted based on the observation of new vessels protruding from the aorta. The percentage of vessels sprouting was determined by comparing number of protruded vessels in DK1 treated group with untreated control group. This study was approved by Institutional Animal Care and Use Committee, Universiti Putra Malaysia (UPM/IACUC/AUP-R001/2018)

### 4.7. Microarray-Based Gene Expression Analysis

Microarray analysis was used to study the gene expression of both U-2 OS and MG-63 human osteosarcoma cell lines treated with curcumin analogue DK1 at IC_50_ for 48 h. For this particular experiment, untreated group of both U-2 OS and MG-63 cell lines were served as control group plus each experimental group was done in triplicate. SurePrint G3 Human Gene Expression 8X60k v3 Microarray kit from Agilent Technologies, Santa Clara, CA, USA was used in this experiment. For starting input, 25 ng of total RNA extracted from the human osteosarcoma cell lines was used to synthesize the cDNA by following the RNA Spike-In Kit manufacturer’s protocol (Agilent Technologies, Santa Clara, CA, USA). Then cRNA was synthesized and labeled with low Input Quick Amp Labelling Kit, one color cyanine 3 from Agilent Technologies, Santa Clara, CA, USA and further purified using RNAeasy Mini Kit from Qiagen, Hilden, NRW, Germany. The concentration of purified cRNA was then measured by using the ND-1000 UV-VIS Spectrophotometer (Agilent Technologies, Santa Clara, CA, USA), this reading can be used to determine the specific activity for each reaction and the yield approximately. Next, the cRNA was then hybridize onto Agilent SurePrint G3 Human GE 8X60K Microarray slide and further incubated at 65 °C, 10 rpm for 17 h in Agilent hybridization oven. Prior to scanning using Agilent SureScan D (G4900DA), the microarray slide needs to be washed with Expression Wash Buffer 1 and 2 (Agilent Technologies, Santa Clara, CA, USA). Finally, to analyze the differential expression between the DK1 treated group and the untreated group, GeneSpring v13 was utilized with necessary statistical filtration fixed at T-test (*p* < 0.05) and fold change > 2. 

### 4.8. Real-time Quantitative PCR Analysis

Total RNA was extracted using QIAGEN RNAeasy Kit (Qiagen, Hilden, NRW, Germany) by following the provided manufacturer’s protocol. The purity and concentration of the isolated RNA was determined using spectrophotometer (Beckman Coulter, Brea, CA, USA), meanwhile the integrity of the isolated RNA was evaluated by running on 1% agarose gel. cDNA was then synthesized using 5 µg of total RNA as starting material using RevertAid First Strand cDNA Synthesis Kit (Thermo Scientific, Waltham, MA, USA). All primer sequences and the accession number that used for the gene expression analysis were provided in Appendix A (Table A1). Thermo Scientific Luminaris Color HiGreen qPCR Master Mix (Thermo Scientific, Waltham, MA, USA) was used to carry out the real-time PCR on the Eco™ Real-Time PCR System (Illumina, San Diego, CA, USA). PCR reaction was initiated at 95 °C for 10 min, followed by denaturation at 95 °C for 10 s and annealing/extension at 58 °C for 30 s. These denaturation and extension phases were repeated for 40 cycles. EcoStudy Software v4.0 (Illumina, San Diego, CA, USA) was used to determine the primer efficiency and to obtain the qPCR results by normalizing the genes expression to two housekeeping genes (ACTB; GUSB). By comparing the normalized expression of untreated group to the DK1-treated group the fold-change values can be calculated. 

### 4.9. Angiogenesis-Related Proteome Profiling

Treatment using IC_50_ concentration of DK1 was used to study the angiogenesis-related protein expression in both human osteosarcoma cell lines. This expression study was performed using Human Angiogenesis Array Kit (R&D Systems, Minneapolis, MN, USA). Prior to perform the proteome analysis, the protein was extracted from human osteosarcoma cell lines by using RIPA buffer (50 mM Tris-HCl, 150 mM NaCl, 1.0% TritonX-100, 0.5% sodium deoxycholate, 0.1% SDS) supplemented with 10 mg of pre-made protease inhibitor cocktails (Roche, Basel, Canada). The extracted protein was then quantified using Bradford assay (Sigma, St. Louis, MO, USA). Next, 500 µL of the extracted protein were mixed with 500 µL of array buffer 4 and 15 µL of detection antibody cocktail before incubated for 1 h at room temperature. Concurrently, the membranes provided in the kit also were incubated for 1 h in 2 mL of blocking buffer placed on a shaker. The mixtures were then added onto the membrane and incubated at 4 °C overnight. After incubation, the membranes were washed three times with 20 mL of 1X wash buffer and subsequently 2 mL of Streptavidin-HRP diluted in array buffer 5 was loaded on the membranes and incubated on shaker for 30 min. The membranes were washed again with 20 mL of 1X wash buffer before exposed to 1 mL of Chemi Reagent Mix for viewing. Lastly, the membrane was analyzed using the ChemiDoc XRS (BioRad, Hercules, CA, USA). Images of the membranes were included in Appendix B.

### 4.10. Statictical Analysis

All data were presented as a statistical mean ± S.E.M (Standard Errors Mean) from three independent experiments. Data with *p* < 0.05 was considered to be statistically significant and one-way ANOVA was utilized to do the statistical comparison. SPSS v20 was used to conduct all statistical analysis.

## 5. Conclusions

This study deduced that curcumin analog DK1 successfully exhibited anti-metastatic and anti-angiogenic capabilities by inhibiting the cell motility, migration/invasion as wells as tube formation and micro-vessels sprouting. These observations were substantially supported when several genes (PLK3, PTEN, FOXO, GADD45A, and Caspase) that related to metastasis inhibition were significantly up-regulated and several other genes and protein associated with promoting the metastasis activity (Endoglin, uPA, Serpine, IGFBP-2, and FGF) were notably down-regulated. Further extensive study needs to be done specifically in toxicity evaluation as well as in vivo osteosarcoma model in order to bolster the DK1 potential as an anti-osteosarcoma agent.

## Figures and Tables

**Table 1:** List of regulated genes, associated with either in promoting cell metastasis or results in inhibitory activity for U-2 OS treated with DK1 (IC_50_) compared with untreated control.

Gene Bank	Gene Symbol	Gene Name	Fold Change > 2
Pro-metastasis			
NM_016653	ZAK	Homo sapiens mitogen-activated protein kinase kinase kinase 20 (MAP3K20)	-3.53
NM_002982	CCL2	Homo sapiens C-C motif chemokine ligand 2	−4.28
NM_002422	MMP3	Homo sapiens matrix metallopeptidase 3	−4.44
NM_000800	FGF1	Homo sapiens fibroblast growth factor 1	−5.23
NM_002006	FGF2	Homo sapiens fibroblast growth factor 2	−3.86
NM_000088	COL1A1	Homo sapiens collagen type I alpha 1 chain	−3.08
NM_001626	AKT2	Homo sapiens AKT serine/threonine kinase 2	−2.30
NM_004655	AXIN2	Homo sapiens axin 2	−2.02
NM_006739	MCM5	Homo sapiens minichromosome maintenance complex component 5	−2.40
NM_001105209	LAMA4	Homo sapiens laminin subunit alpha 4	−8.16
NM_033131	WNT3A	Homo sapiens Wnt family member 3A	−4.23
NM_001303429	PIK3R3	Homo sapiens phosphoinositide-3-kinase regulatory subunit 3	−6.54
Metastasis Inhibition			
NM_020529	NFKBIA	Homo sapiens NFKB inhibitor alpha	2.50
NM_004556	NFKBIE	Homo sapiens NFKB inhibitor epsilon	2.24
NM_005427	TP73	Homo sapiens tumor protein p73	3.41
NM_078467	CDKN1A	Homo sapiens cyclin dependent kinase inhibitor 1A	5.03
NM_000314	PTEN	Homo sapiens phosphatase and tensin homolog	2.14
NM_005194	CEBPB	Homo sapiens CCAAT enhancer binding protein beta	2.65
NM_014417	BBC3	Homo sapiens BCL2 binding component 3	3.08
NM_004073	PLK3	Homo sapiens polo like kinase 3	7.29
NM_170662	CBLB	Homo sapiens Cbl proto-oncogene B	2.23
NM_001455	FOXO3	Homo sapiens forkhead box O3	2.77
NM_001924	GADD45A	Homo sapiens growth arrest and DNA damage inducible alpha	3.92
NM_004292	RIN1	Homo sapiens Ras and Rab interactor 1	2.77
NM_004418	DUSP2	Homo sapiens dual specificity phosphatase 2	4.76
NM_007207	DUSP10	Homo sapiens dual specificity phosphatase 10	4.95

Notes: All differential regulated genes were expressed in fold changes ≥ 2or ≤ −2, *p* < 0.05 compared with corresponding control.

**Table 2:** Summary of regulated genes, associated with either in promoting cell metastasis or results in inhibitory activity for MG−63 treated with DK1 (IC_50_) compared with untreated control.

Gene Bank	Gene Symbol	Gene Name	Fold Change > 2
Pro-metastasis			
NM_000602	SERPINE1	Homo sapiens serpin family E member 1	−8.96
NM_001025370	VEGFA	Homo sapiens vascular endothelial growth factor A	−2.67
NM_003377	VEGFB	Homo sapiens vascular endothelial growth factor B	−2.56
NM_001626	AKT2	Homo sapiens AKT serine/threonine kinase 2	−3.90
NM_000657	BCL2	Homo sapiens BCL2 apoptosis regulator	−3.54
NM_001798	CDK2	Homo sapiens cyclin dependent kinase 2	−4.68
NM_001556	IKBKB	Homo sapiens inhibitor of nuclear factor kappa B kinase subunit beta	−2.23
NM_014002	IKBKE	Homo sapiens inhibitor of nuclear factor kappa B kinase subunit epsilon	−3.77
NM_000597	IGFBP2	Homo sapiens insulin like growth factor binding protein 2	−2.33
NM_002009	FGF7	Homo sapiens fibroblast growth factor 7	−8.49
NM_001105209	LAMA4	Homo sapiens laminin subunit alpha 4	−5.96
NM_001010931	HGF	Homo sapiens hepatocyte growth factor	−10.78
Metastasis Inhibition			
NM_002015	FOXO1	Homo sapiens forkhead box O1	4.03
NM_018947	CYCS	Homo sapiens cytochrome c	2.82
NM_001229	CASP9	Homo sapiens caspase 9	2.14
NM_004346	CASP3	Homo sapiens caspase 3	2.16
NM_001924	GADD45A	Homo sapiens growth arrest and DNA damage inducible alpha	8.90
NM_015999	ADIPOR1	Homo sapiens adiponectin receptor 1	2.54
NM_170662	CBLB	Homo sapiens Cbl proto-oncogene B	5.41
NM_006705	GADD45G	Homo sapiens growth arrest and DNA damage inducible gamma	8.40
NM_005359	SMAD4	Homo sapiens SMAD family member 4	2.46
NM_000314	PTEN	Homo sapiens phosphatase and tensin homolog	2.34
NM_004073	PLK3	Homo sapiens polo like kinase 3	5.36
NM_001040619	ATF3	Homo sapiens activating transcription factor 3	13.86
NM_004418	DUSP2	Homo sapiens dual specificity phosphatase 2	2.27
NM_007207	DUSP10	Homo sapiens dual specificity phosphatase 10	5.27

Notes: All differential regulated genes were expressed in fold changes ≥ 2 or ≤ −2, *p* < 0.05 compared with corresponding control.

**Table 3:** Metastasis and angiogenesis related proteins expression in DK1 treated group for both U-2 OS and MG-63 osteosarcoma cell lines.

Cell Lines	Proteins	Relative Intensity (Fold Change)	Regulation
**U-2 OS**	Endoglin	−7.6 * ± 0.04	Down
	uPA	−2.4 * ± 0.04	Down
	IGFBP-2	−1.7 * ± 0.07	Down
	FGF	−1.4 * ± 0.01	Down
	Serpin F1	−1.4 * ± 0.05	Down
	IGFBP-1	−1.5 * ± 0.06	Down
	Serpin E1	1.7 * ± 0.13	Up
	TGF-ꞵ1	−1.4 * ± 0.04	Down
**MG-63**	Serpin E1	−3.6 * ± 0.02	Down
	VEGF	−2.0 * ± 0.01	Down
	uPA	−3.3 * ± 0.04	Down
	Endoglin	−12.5 * ± 0.01	Down
	IGFBP-2	−6.2 * ± 0.13	Down
	FGF-7	−3.2 * ± 0.12	Down
	PD-ECGF	−4.7 * ± 0.01	Down
	Endostatin	−1.9 * ± 0.07	Down

Notes: Both osteosarcoma cell lines were treated with DK1 (IC_50_) for 48 h. All data were expressed as mean ± standard error mean (S.E.M) and the significant of the data was set at * *p* < 0.05 compare with corresponding control. (-) the symbol was designated for protein with down-regulated expression.

**Figure A1:**
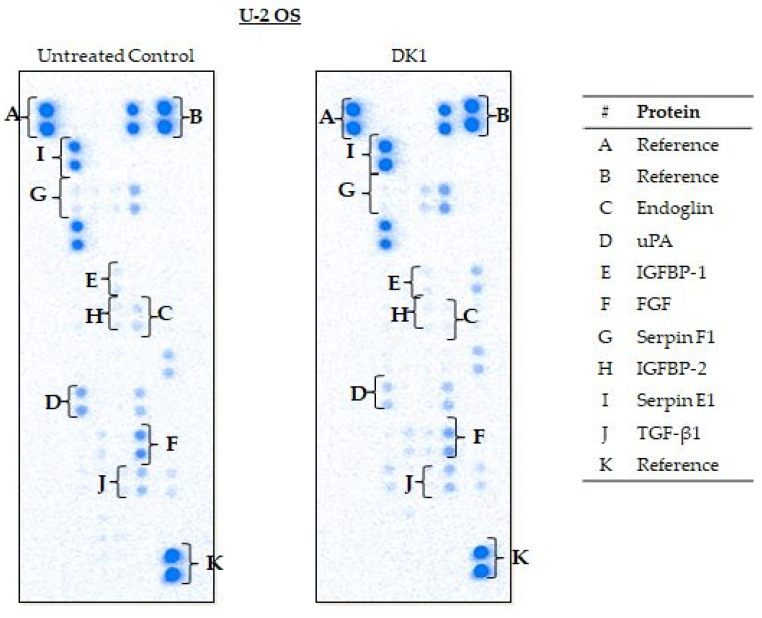
The proteome profiler membrane that exhibits the expression of metastasis-related proteins between untreated control and curcumin analog DK1 in U-2 OS human osteosarcoma cell line.

**Figure A2:**
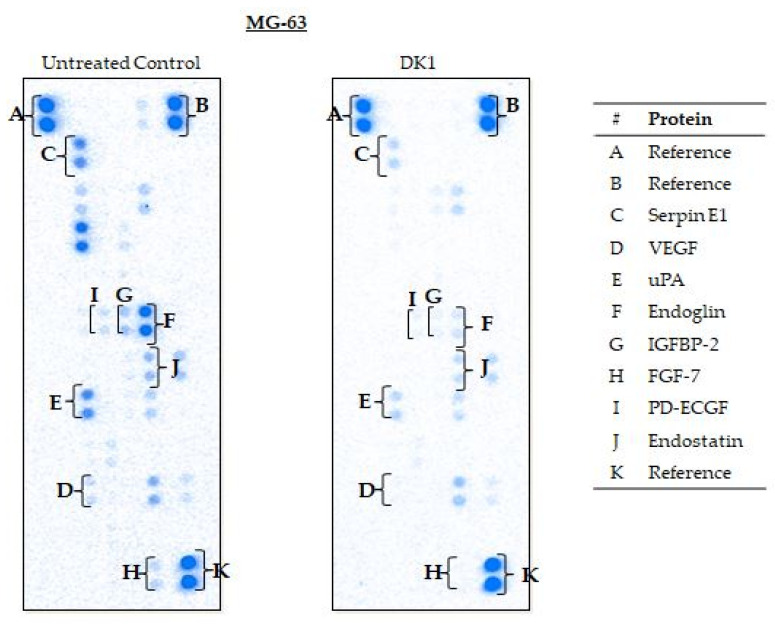
The proteome profiler membrane that manifests the expression of metastasis-related proteins between untreated control and curcumin analog DK1 in MG-63 human osteosarcoma cell line.

## Data Availability

The data presented in this study are available within the article.

## Appendix A

**Table A1:** The primer sequence used in quantitative real-time PCR (qPCR) analysis.

Genes	Accession Number	Forward Primers	Reverse Primers	Amplicon Size (bp)	Primer Efficiency (%)
NFKBIA	NM_020529	GTTACCTACCAGGGCTATTCTC	CTGTGAACTCCGTGAACTCT	163	98
NFKBIE	NM_004556	GAAGCACTCACTTACATCTC	GCTGTCTGGTAAAGGTTATT	143	105
TP73	NM_005427	AGCAGCCCATCAAGGAGGAGTT	TCCTGAGGCAGTTTTGGACACA	132	95
CDKN1A	NM_078467	GGGGTTATCTCTGTGTTAGGG	AGCCTCTACTGCCACCATCT	179	101
PTEN	NM_000314	CACTTGTGGCAACAGATAAG	GTCCAGAGTCCAGCATAAA	138	110
MMP3	NM_002422	AAGATGCTGTTGATTCTGCTGT	ATTGGTCCCTGTTGTATCCTTT	211	110
COL1A1	NM_000088	TCTGCGACAACGGCAAGGTG	GACGCCGGTGGTTTCTTGGT	146	103
FGF1	NM_000800	TGAGAAGAAGACACCAAGTGGA	TTGTGGCGCTTTCAAGACTA	110	95
CDK1	NM_001170406	CAGACTAGAAAGTGAAGAGGAAGG	ACTGACCAGGAGGGATAGAATC	191	97
Cycs	NM_018947	ACCTTCCATCTTGGCTAGTTGTG	ATCGCTTGAGCCTGGGAAATAG	129	100
Casp 3	NM_004346	AGAACTGGACTGTGGCATTGAG	GCTTGTCGGCATACTGTTTCAG	191	103
Casp 9	NM_001229	CTTCGTTTCTGCGAACTAACAGG	GCACCACTGGGGTAAGGTTT	75	106
SERPINE1	NM_000602	ACTTTTCAGAGGTGGAGAGA	CCAGGATGTCGTAGTAATGG	300	107
IKBKE	NM_014002	GTCAAACGACCTCATCACA	CCAAGCATAGAAAGAACAGG	142	106
AKT2	NM_001626	ACCTCTGGGTGTTTGGAGTT	CCCTGGTCTGAAATGAGTGT	231	107
BCL2	NM_000657	TTGACAGAGGATCATGCTGTACTT	ATCTTTATTTCATGAGGCACGTT	103	110
VEGFA	NM_001025370	AGGGCAGAATCATCACGAAGT	AGGGTCTCGATTGGATGGCA	75	105
IKBKB	NM_001556	CTACTTTGGTGGTTGTCCTC	ATACCCTTTCTGTCCCTCCT	176	107
GUSB	NM_000181.4	CACCAGGGACCATCCAATACC	GCAGTCCAGCGTAGTTGAAAAA	99	103
ACTB	NM_001101.3	AGAGCTACGAGCTGCCTGAC	AGCACTGTGTTGGCGTACAG	184	110
